# Genetic Functionalization of Protein-Based Biomaterials via Protein Fusions

**DOI:** 10.1021/acs.biomac.4c00188

**Published:** 2024-07-29

**Authors:** Gabriela Geraldo Mendes, Britt Faulk, Bhavika Kaparthi, Andrew R. Irion, Brandon Look Fong, Kayla Bayless, Sarah E. Bondos

**Affiliations:** † Department of Molecular and Cellular Medicine, College of Medicine, Texas A&M Health, Bryan, Texas 77807-3260, United States; ‡ Fralin Biomedical Research Institute, Virginia Tech University, Roanoke, Virginia 24016, United States; § Department of Medical Physiology, College of Medicine, Texas A&M Health, Bryan, Texas 77807, United States; ∥ Department of Biochemistry and Biophysics, Texas A&M University, College Station, Texas 77843, United States; ⊥ Department of BioSciences, Rice University, Houston, Texas 77005, United States

## Abstract

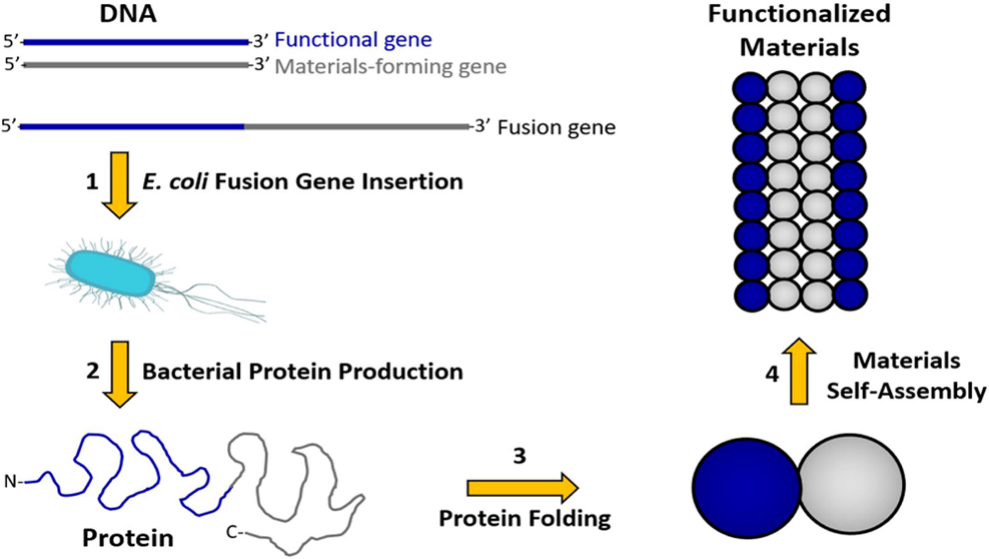

Proteins implement many useful functions, including binding ligands with unparalleled affinity and specificity, catalyzing stereospecific chemical reactions, and directing cell behavior. Incorporating proteins into materials has the potential to imbue devices with these desirable traits. This review highlights recent advances in creating active materials by genetically fusing a self-assembling protein to a functional protein. These fusion proteins form materials while retaining the function of interest. Key advantages of this approach include elimination of a separate functionalization step during materials synthesis, uniform and dense coverage of the material by the functional protein, and stabilization of the functional protein. This review focuses on macroscale materials and discusses (i) multiple strategies for successful protein fusion design, (ii) successes and limitations of the protein fusion approach, (iii) engineering solutions to bypass any limitations, (iv) applications of protein fusion materials, including tissue engineering, drug delivery, enzyme immobilization, electronics, and biosensing, and (v) opportunities to further develop this useful technique.

## 1 Introduction

By forming multiple weak bonds with ligands, proteins can reversibly bind specific molecules with very high affinity. *In vivo*, proteins require these traits to create and regulate the vital chemical pathways, cells, and tissues necessary for life. The wide variety of natural protein functions has been further augmented by engineering proteins with novel abilities.^1,2^ Embedding active proteins in materials imparts these efficient and specific functions for use in many devices, including biomolecular sensors,^3,4^ drug delivery,^5−7^ chemical catalysis,^8^ optics and electronics,^9,10^ and tissue engineering.^4,9−19^

Creating devices using materials composed of proteins confers many advantages, including providing a facile method to stabilize useful proteins by embedding them into the materials. The fidelity of transcription and translation (1 error per 10^4^ amino acids) ensures that each protein has the same length and amino acid sequence.^20,21^ Thus, proteins produced from a single gene in a constant environment will have the same three-dimensional structure and stability, and, for proteins that form higher-order complexes, the same ability to assemble.^20−22^ Protein-based biomaterials can have useful mechanical properties, including extreme strength, as exemplified by dragline silk, and high extensibility, as observed for elastin and elastin-like proteins (ELPs). Finally, the sequence of the protein monomers can be easily and quickly engineered using standard molecular biology techniques, allowing the materials’ sequence, assembly mechanism, structure, stability, and physical properties to be tailored for specific applications.^23^

As a normal component of the body, proteins are generally biodegradable and nontoxic, with some exceptions such as toxins used by animals and plants for defense.^19,24−29^ Although protein monomers can elicit antibody production when injected into an animal host, assembly of these monomers into a stable material tends to prevent an immune response. Indeed, many recombinant materials-forming proteins produced in bacteria or insects are biocompatible and nonimmunogenic.^30−32^ Consequently, protein-based materials are often suitable for *in vivo* applications. While recombinant protein production in *Escherichia coli* confers many advantages, including low cost and ease of fermentation, these proteins can copurify with toxic bacterial endotoxins, which must be removed before their application in human therapies.^33−35^ To solve this problem, a broad range of organisms are being used as cell factories as an alternative to *E. coli*, including a recently developed strain of *E. coli* that only produces a truncated endotoxin, other types of bacteria, yeast, insect cells, and mammalian cells (especially Chinese hamster ovary cells), among other production systems.^18−20^

## 2 Advantages of Fusion Proteins

**Figure 1:**
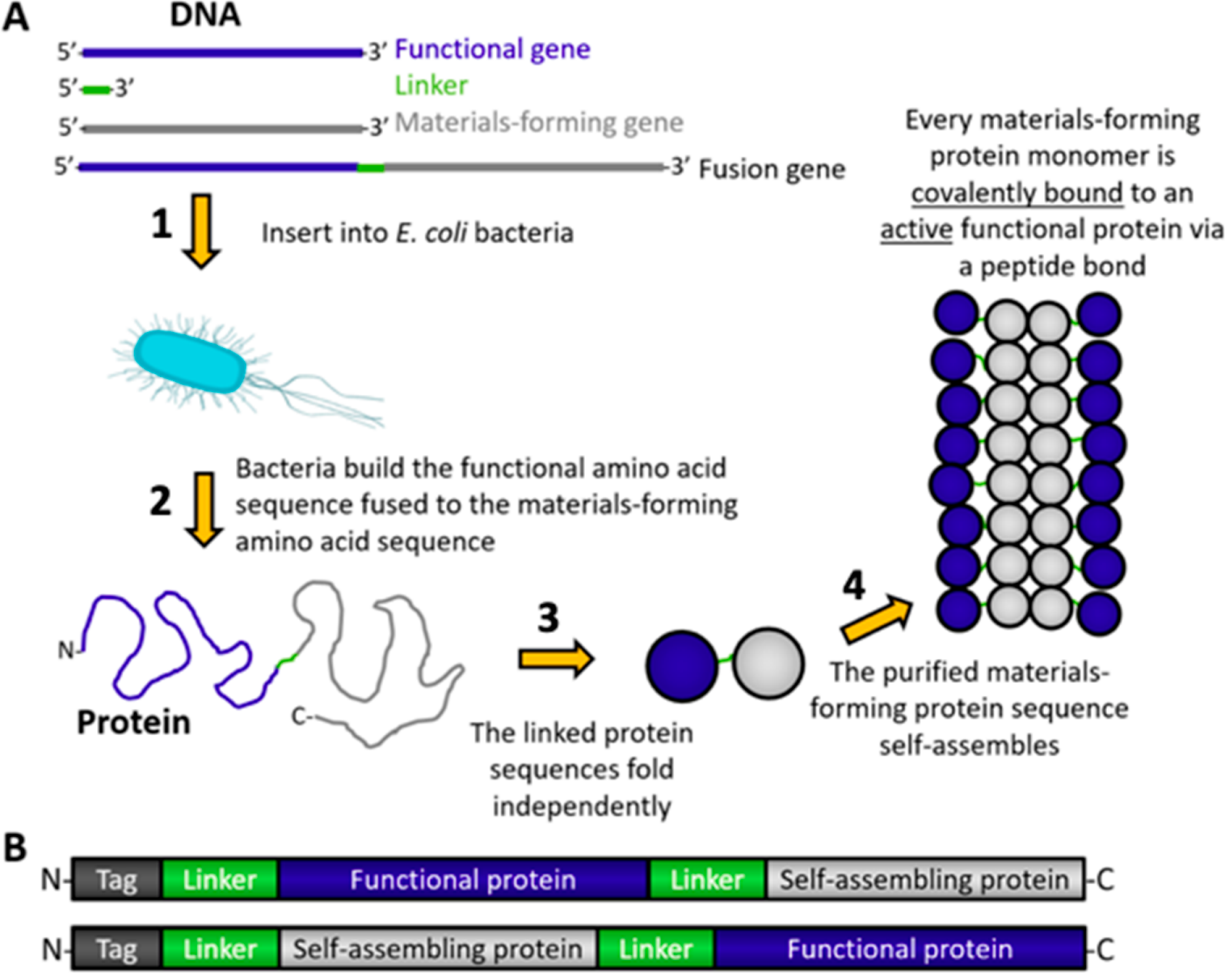
Fusion proteins in materials assembly. A) A schematic depicting the production of fusion proteins and their assembly into materials. B) Schematic of fusion protein sequences. The functional protein (dark blue) may be placed N-terminal (top) or C-terminal (bottom) to the self-assembling protein (gray) or within a flexible loop of the self-assembling protein. Purification tags (dark gray) may be included at either terminus or within the protein sequence. Soluble, flexible linkers (green) are often required to prevent each component from sterically interfering with the folding or function of other components of the fusion protein.

Materials that are composed of proteins offer a unique opportunity to efficiently immobilize and display useful functional proteins. Facile molecular biology tools can be used to produce both functional and self-assembling proteins as a single linear chain of amino acids linked by a covalent peptide bond (Figure 1).^19,36−39^ The sequence of amino acids is unique to each protein and genetically encoded in the sequence of nucleic acids in that protein’s gene (DNA), which is also a linear sequence. To create a “fusion gene”, the DNA sequence encoding the functional protein is placed end-to-end with the DNA sequence encoding the self-assembling protein, without intervening stop codons. When the appropriate regulatory DNA sequences are added to the fusion gene and placed in a live cell (Figure 1, Step 1), the cell will use the DNA instructions to produce a single “fusion protein” that contains the amino acid sequence of both proteins in a single chain of amino acids (Figure 1, Step 2). In the example shown in Figure 1 (top), the functional protein sequence is followed by the sequence of the self-assembling protein. Ideally, these proteins will independently fold into their unique 3-dimensional structures defined by their amino acid sequence (Figure 1, Step 3), and the self-assembling protein portion of the fusion protein will interact with other copies of the fusion protein to form materials. The structure and function of the fused functional proteins are often preserved and improved due reduced protease access and limited mobility. This confined space surrounding the fused proteins hampers protein unfolding, resulting in prolonged enzymatic, signaling, and/or biological activities.. In practice, an additional short linker sequence of amino acids must often be added in between the functional sequence and the self-assembling sequence to prevent one sequence from sterically hindering the folding or function of the other sequence. This approach can be used to incorporate peptides, protein domains, and full-length, folded proteins into a wide variety of protein-based materials (Tables 1 and 2).

**Table 1:** A Representative Subset of Protein Domains Genetically Fused to Self-Assembling Proteins That for Macroscale Materials Is Listed^a^

fusion protein	functional peptide or domain added	self-assembling protein	linker	fusion terminus	ref
Ligand-Binding Protein or Domain Added					
FN-Ubx	Type III domain 8–10 of Fibronectin (FN)	Ultrabithorax (Ubx)	GH	N	(36)
Z-4RepCT	IgG-binding domain Z	4RepCT^b^	LEALFQGPNS	N	(39)
C2-4RepCT	IgG-binding domain C2	4RepCT^b^	LEALFQGPNS	N	(39)
ABD-4RepCT	Albumin-binding domain ABD	4RepCT^b^	LEALFQGPNS	N	(39)
M4-4RepCT	Biotin-binding domain M4	4RepCT^b^	GNS	N	(39)
CABP	Fragment from Calcium-binding protein (CABP)^b^^,^^d^	Fibroin light chain	None	C	(37)
6mer + FNII	Fibronectin Type II (FNII) module	6mer^b^	None	C	(40)
BC-MAP	B and C domains of protein A	Mussel adhesive protein	PWGGGG	N	(41)
RLP12	RGDSP (cell binding peptide), GPQGIWGQ (protease cleavage site), and CKAAKRPKAAKDKQTK (heparin binding domain)	12 resilin-like motifs	GGKGG, AEDL, and GGRGG	Middle	(42)
VE-M	VE-cadherin extracellular domain EC1-2	Mussel foot protein (Mfp-5)	GGGGS	N	(43)
ScFv-NC	Single-chain variable fragments	N- and C-terminal domains of natural spider silk	None	N	(44)
Fn3-ELP (BC)	Fibronectin type III domain	ELP^c^	None	N	(45)
Knob-ELP	Adenovirus Knob domain	ELP^c^	GLVPRGS	N	(46)
FibLanti-WASP-scFv	Single-chain variable mAb fragments	Silkworm silk	None	C	(47, 48)

a Linker refers to the 1 letter code for amino acids inserted between the functional protein domain and the self-assembling protein. Fusion Terminus indicates whether the functional protein was fused to the N-terminal side, the C-terminal side, or in the middle of the self-assembling protein.

b Produced in *Bombyx mori* instead of *E. coli*.

c Elastin-like polypeptide (ELP).

d Silkworm silk derivative.

**Table 2:** Full-Length Proteins Incorporated into Macroscale Protein-Based Materials as Protein Fusions^a^

N	functional protein added	self-assembling protein	linker	fusion terminus	ref
Fluorescent or Luminescent Protein Added					
L-chain-EGFP fusion protein^b^	Enhanced Green Fluorescent Protein (EGFP)	Light chain^d^	None	C	(49)
mCherry-Ubx	mCherry	Ubx	GH	N	(38)
EGFP-Ubx	EGFP	Ubx	GH	N	(38)
AmCyan-Ubx	AmCyan	Ubx	GH	N	(36)
EBFP-Ubx	Enhanced Blue Fluorescent Protein (EBFP)	Ubx	GH	N	(36)
BFP-ELP	Blue Fluorescent Protein (BFP)	ELP^c^	None	N	(50)
A-mCherry-A	mCherry	ELP/SpyTag (AA) + ELP/SpyCatcher (BB) mix^c^	Not specified	Middle	(51)
HUG	UnaG	Human ELP^c^	None	C	(52)
V50/GFP	Superfolder GFP	V50 ELP^c^	VPGGG	C	(10)
V50/BFP	mTurquoise2	V50 ELP^c^	VPGGG	C	(10)
V50/YFP	sYFP2	V50 ELP^c^	VPGGG	C	(10)
V50/RFP	RFP	V50 ELP^c^	VPGGG	C	(10)
Ligand-Binding Protein Added					
Myoglobin-Ubx	Myoglobin	Ubx	Gly-His	N	(38)
MBP-Ubx	Maltose-binding protein	Ubx	GTNIDDDDKHMSGSG	N	(36)
TneSSB-Ubx	*Thermotoga neapolitana* single-stranded DNA binding protein	Ubx	GSGSH	N	(36)
TmaSSB-Ubx	*Thermatoga maritime* single-stranded DNA binding protein	Ubx	GSGSH	N	(36)
N-WT–CspB	Cold-shock protein from *Bacillus subtilis* (CspB)	N-terminal domain of PABPN1	GAAGG	C	(53)
N-(+7)Ala-CspB	CspB	N-terminal domain of PABPN1	GAAGG	C	(53)
N-ΔAla-CspB	CspB	N-terminal domain of PABPN1	GAAGG	C	(53)
hGal3–4RepCT	Galectin-3 (mucin binding)	4RepCT^e^	Not Specified	N	(5)
ELP-sTNFRII	Cleavage product of Soluble Tumor Necrosis Factor Receptor II	ELP^c^	None	C	(54)
Fp-151-VT	Vitronectin	Mussel Foot Protein variant	KL	C	(55)
CypA-ELP	Cyclosporine A	(VPGAG)_192_Y^c^	None	N	(56)
FAF	FKBP12	(VPGAG)_192_Y^c^	G (N-terminal fusion only)	N & C	(57)
ELP-IL-1Ra	Interleukin-1 Receptor Antagonist	ELP^c^	None	C	(58)
Enzyme Added					
Xyl-4RepCT	Xylanase	4RepCT^4^	PNS	N	(59)
Xyl-CT	Xylanase	C-terminal domain^4^	PNSGIQ	N	(59)
GST-Ubx	Glutathione S-transferase	Ubx	GTNIDDDDKHMSGSG	N	(36)
PFK-Ubx	Phosphofructokinase	Ubx	GSGSH	N	(36)
L-PYK-Ubx	Liver pyruvate kinase	Ubx	GGSGSH	C	(36)
NusA-Ubx	N utilization substance protein A	Ubx	GTNIDDDDKHMSGSG	N	(36)
Luciferase-Ubx	Luciferase	Ubx	GH	N	(38)
Thioredoxin-Ubx	Thioredoxin	Ubx	GTNIDDDDKHMSGSG	N	(36)
Trx-ELP	Thioredoxin	ELP^c^	SSGLVPRGS	N	(50)
Trx-ELP & ELP-Trx	Thioredoxin	ELP^c^	None	N or C	(50)
CAT-ELP & ELP-CAT	Chloramphenicol acetyltransferase (CAT)	ELP^c^	None	N or C	(50)
NusA-NusA-Ubx	N utilization substance protein A	Ubx	GTNIDDDDKHMSGSG	N	(36)
HS-OPH	Organophosphate hydrolase (OPH)	α-helical leucine zipper domains (H)	soluble linker domains (S), (AGAGAGPEG)_10_	N	(60)
HS-OPH-H	Organophosphate hydrolase (OPH)	α-helical leucine zipper domains (H)	soluble linker domains (S), (AGAGAGPEG)_10_	N & C	(60)
6His-HS-OPH	Organophosphate hydrolase (OPH)	α-helical leucine zipper domains (H)	soluble linker domains (S), (AGAGAGPEG)_10_	N	(60)
6His-HS-OPH-H	Organophosphate hydrolase (OPH)	α-helical leucine zipper domains (H)	soluble linker domains (S), (AGAGAGPEG)_10_	N & C	(60)
HS-Adh-H	Alcohol dehydrogenase	α-helical leucine zipper domains (H)	soluble linker domains (S), (AGAGAGPEG)_10_	Middle	(61)
BGlucEH	β-Glucosidase	ELP^c^	GGGGSGGGGSGGGGS	N	(62)
Growth Factor/Cytokine Added					
r(FL/bFGF)^b^	Basic Fibroblast Growth Factor (bFGF)	Light Chain^3^	None	C	(63)
SDF-1α-Ubx	Stromal cell-derived factor-1α (SDF-1α)	Ubx	Gly-His	N	(36)
bFGF-Ubx	bFGF	Ubx	Gly-His	N	(36)
4RepCT-bFGF	bFGF	4RepCT^e^	Gly-Ser	C	(64)
VEGF-Ubx	Vascular endothelial growth factor (VEGF)	Ubx	Gly-His	N	(36)
VEGF-ELP	VEGF	ELP^c^	Gly-Ser-Gly	C	(65)
Osteopontin- Ubx	Osteopontin	Ubx	Gly-His	N	(36)
6mer + BSP	Bone sialoprotein (BSP)	6mer^e^ silk	Not specified	C	(66)
A-LIF-A	MH35-LIF, a mouse/human Leukemia Inhibitory Factor chimera	ELP/SpyTag (AA) + ELP/SpyCatcher (BB) mix^c^	Not specified	Middle	(51)
FibH-hEGF	Human Epidermal Growth Factor	Fibroin Heavy Chain^d^	None	C	(67)
IFN-ELP	Interferon	ELP^c^	None	N	(68, 69)
RGD-TRAIL-ELP	Tumor Necrosis Factor-related Apoptosis-Inducing Ligand	ELP^c^	None	N	(70)
PDGF-BB sericin	Platelet Derived Growth Factor B fused C-terminally to a domain of placental growth factor 2	Sericin^d^	Not Specified	N	(71, 72)
FibH-EGF	Endothelial Growth Factor	Truncated Fibroin Heavy chain^d^	None	C	(73)
hGMCSF-ELP	Human Granulocyte	(VPGAG)_192_Y^c^	G	N	(74)
KGF-ELP	Keratinocyte Growth Factor (aka FGF7)	(VPGVG)_120_^c^	None	N	(75)
Receptor or Receptor Binding Protein Added					
Di-Z_VEGFR2-S_-Silk	di-Z_VEGFR2_, a VEGFR2-specific dimeric affibody molecule	4RepCT^e^	None	N	(76)
Tetra-Z_VEGFR2-L1-S_-Silk	tetra-Z_VEGFR2_, VEGFR2-specific tetrameric affibody molecule	4RepCT^e^	None	N	(76)
IL1Ra-ELP	Interleukin-1 Receptor Antagonist	ELP^c^	None	N	(50)
ELP-IL1Ra	Interleukin-1 Receptor Antagonist	ELP^c^	None	C	(50)
Regulatory Protein Added					
SUMO-Ubx	Small Ubiquitin-like Modifier Protein	Ubx	GTNIDDDDKHMSGSG	N	(36)
CAV1-ELP	Caveolin	ELP^c^	Myc tag	N	(77)
Mfp-AFP	10 repeats of the motif “A/TCTxSxxCxxAx” derived from Antifreeze Protein	5 repeats of the Mussel Foot Protein motif “YKYKV”	None	C	(78)
Azurin-ELP	Azurin, a p53 regulator	ELP	None	N	(79, 80)
Two or More Functional Proteins, Domains, or Peptides Added					
GEF	Fibroblast Growth Factor 21	ELP^c^	GGGGSGGGGSGGGGS	N & C	(81)
	Glucagon-like Peptide 1				
EGFP-VEGF-Ubx	EGFP and VEGF	Ubx	Gly-His	N	(12)
ELP-DCN	Poly aspartic acid, IgG, NanoLuciferase	ELP^c^	Not specified	C	(82)
KTP-ELF-VEGF	Kidney Targeting Peptide And VEGF	ELP^c^	None	N & C	(83)

a Linker refers to the 1 letter code for amino acids inserted between the functional protein domain and the self-assembling protein. Fusion Terminus indicates whether the functional protein was fused to the N-terminal side, the C-terminal side, or in the middle of the self-assembling protein.

b Produced in *Bombyx mori* instead of *E. coli*.

c ELP, elastin-like protein.

d Silkworm silk derivative.

e Spider silk derivative.

There are numerous advantages to this approach. By combining production of the functional protein, production of the self-assembling protein, and protein immobilization into a single-step, single-pot process, the time and cost required to produce materials is substantially reduced.^84,85^ Therefore, the extent of functionalization does not have to be independently validated after each production batch. The added functional protein has the potential to increase the yield of the recombinant fusion protein, an effect that has been observed in *E. coli*(36,40) and in the silkworm *Bombyx mori*.^86^ This increase in yield correlates with the solubility of the fusion protein^36^ and can help prevent the random (nonproductive) association of self-assembling monomers into aggregates.^47^ The functional proteins are uniformly attached to the material by the same bond. Consequently, there are no variations in the attachment site that have the potential to misposition or inactivate a fraction of the functional proteins. Because the functional protein is attached to the material via a covalent peptide bond, any solution conditions that are safe for the materials will also preserve this bond, reducing or eliminating leaching.^87^ Because every monomer has one functional protein attached, by definition the materials are saturated and the coverage is even. For some combinations of functional protein and self-assembling proteins, fusion dramatically increases the stability of the functional protein.^36,39^ However, this is not the case for all fusion proteins that form materials.^88^ Finally, many different proteins can be incorporated and even patterned into a single material as protein fusions.^38^

**Figure 2:**
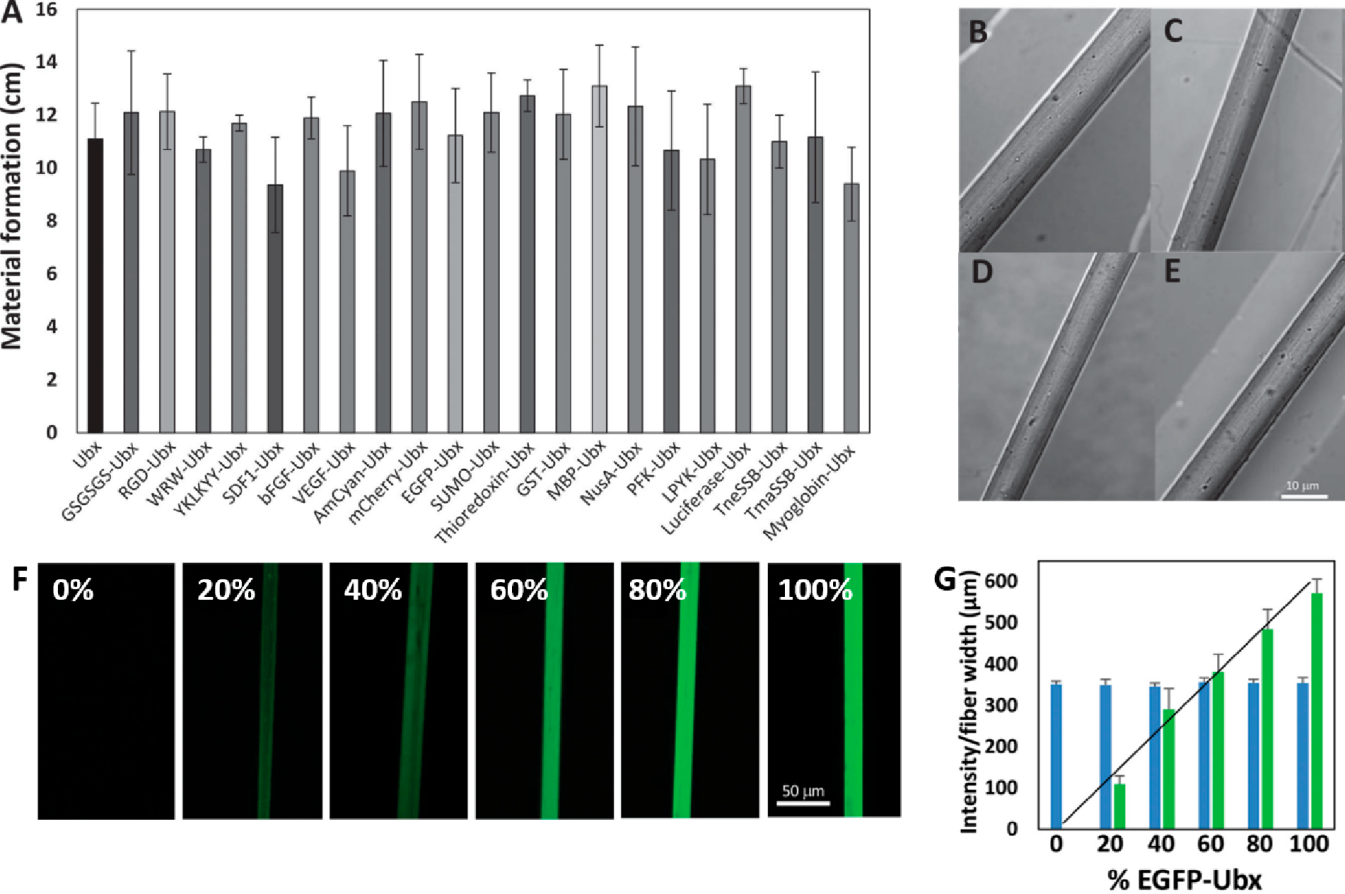
Protein fusion does not significantly impact Ubx assembly into materials. A) Comparison of Ubx materials formation at room temperature for 16 h for unfused Ubx (left) and multiple Ubx peptide and protein fusions. The monomer concentration was 50 nM in all samples. Materials formation was quantified as the length of fiber drawn from the assembly solution, averaged over three experiments.^36^ B–E) Differential interference contrast microscopy of Ubx, VEGF-Ubx, MBP-Ubx, and PFK-Ubx fibers, respectively, demonstrates that protein fusions do not alter the structure of Ubx fibers even when the appended protein forms dimers (VEGF-Ubx) or tetramers (PFK-Ubx).^36^ F) Fibers formed from mixtures of Ubx and EGFP-Ubx fluoresce to different extents. The percent of EGFP-Ubx in each mixture is labeled on each panel. G) Fiber fluorescence (green bars) scales linearly with the percent of EGFP-Ubx in mixture of monomers, indicating neither Ubx nor EGFP-Ubx assembles better than the other protein. To account for differences in fiber size, the intensity of green fluorescence was normalized to fiber width. Blue bars represent the normalized blue fluorescence from dityrosine bonds present in all Ubx materials.^90^ Reprinted with permission from Tsai et al., 2015.^36^

In many cases, the addition of a functional protein does not harm either monomer production or materials assembly.^36,39,44,59^ For instance, the *Drosophila melanogaster* protein Ultrabithorax (Ubx) self-assembles *in vitro* into nanoscale fibrils which further associate into macroscale films and fibers.^89^ Ubx can be fused to much larger proteins, to highly charged proteins, or to dimers/tetramers without impacting its ability to form materials or the morphology of the resulting fibers (Figure 2A,E; Table 2).^36^ Additional evidence that materials formation is unaffected is provided by competing Ubx with Enhanced Green Fluorescent Protein-Ubx (EGFP-Ubx) monomers prior to materials assembly. If EGFP impaired Ubx assembly, then the fraction of Ubx protein in the material should be higher than the fraction of Ubx in the monomer mixture. Instead, the ratio of Ubx and EGFP-Ubx proteins in the final material was the same as in the initial protein mixture. Therefore, both proteins assemble equally well (Figure 2F,G).^36^

The functionality of the appended functional protein is often preserved in protein materials.^36,91,92^ Incorporating functional proteins into materials can stabilize these proteins relative to the corresponding free protein monomer. For example, both EGFP and the catalytic domain of Arginine Deiminase retain activity when fused to human Ferritin heavy chain (hFTN-H), creating the recombinant proteins termed hFTN-H::GFP and hFTN-H::ADI_132–410_. When compared to recombinant EGFP or arginine deiminase monomers, these materials demonstrated increased stability in fluorescence-emitting and arginine-degrading activity upon heating (70 °C) or in the presence of a high concentration of guanidinium hydrochloride (4.5 M), acid, or alkali.^91^ Unlike mCherry monomers, some fluorescence intensity from mCherry is retained in mCherry-Ubx fibers even after ethanol treatment for 30 min (80%) or autoclaving for 20 min (20%), a trait that facilitates materials sterilization.^36^ This increase in stability can assist with materials storage as well. Thatikonda et al. reported that portions of an antibody fused to an engineered spider silk protein (scFv-NC silk fusion protein) created fibers that remained functional after storage at room temperature for 1 week.^44^ Similarly, the enzyme xylanase was 40% active after 11 months of wet storage, > 1 month of dry storage, or 70% ethanol treatment.^59^ The enzyme β-Glucosidase is only 20% active at 60 °C, whereas β-glucosidase fused to an elastin-like polypeptide is 90% active at this temperature.^62^ The source of this incredible stability is likely due to the confined geometry effect, in which functional proteins embedded within the materials lack the space required for the large-scale motions associated with protein unfolding.^92−94^ Specific interactions between the functional and self-assembling proteins may also contribute in some cases.

Finally, more than two genes may be fused, thus incorporating multiple proteins and functions into the same polypeptide. One example of a multifusion protein is resilin–elastin–collagen chimeric recombinant materials, designed to tailor the mechanical properties of the resulting materials.^95^ The EGFP-VEGF-Ubx double fusion also self-assembled into materials, which retained both green fluorescence and VEGF activity.^12^ The addition of EGFP to VEGF-Ubx improved the solubility, and thus protein yield, during monomer production in *E. coli*.^12^ Surprisingly, VEGF was active when positioned as the middle protein in a double fusion protein that is incorporated into materials, despite its requirement for both a surface-exposed dimer interface and a receptor binding interface to function.^12,96^

## 3 Fusion Protein Design, Typical Difficulties, and Solutions

The general design strategies for creating fusion proteins from a self-assembling protein and a functional protein are depicted in Figure 1B. Depending on the proteins used, the functional protein may either be located N-terminal or C-terminal to the self-assembling protein, or even inserted into a loop of the self-assembling protein. Some fusions may need a linking amino acid sequence connecting the functional protein to the self-assembling protein to provide enough space for both proteins to independently fold and function. Usually, these linkers combine highly soluble amino acids with glycine to provide flexibility. A tag may also be included to aid protein purification. This tag may be attached to the N- or C- terminus of either the functional protein or the self-assembling protein. An additional flexible linker is often used to prevent interference between the tag and the adjacent protein.

### 3.1 Functional Proteins

Although protein fusion offers a facile mechanism to functionalize materials, surprisingly few proteins have been incorporated into materials via protein fusion.

#### 3.1.1 Breadth of Proteins Successfully Fused to Materials

Table 1 lists domains, or folded portions of proteins, that have been incorporated into protein materials via protein fusion. These sections of proteins can fold and function independent of the remainder of the protein sequence. Because proteins domains are small, they are less likely to impede materials assembly and thus are often selected for materials functionalization. This approach is often selected for incorporating ligand binding or enzyme catalysis activities. To our knowledge, Table 2 lists all reported full-length proteins incorporated into materials via protein fusions as of January 2024. The most common functions added to materials are fluorescence, ligand binding, enzymatic catalysis, cell binding, and cell signaling (Tables 1, 2). For instance, Myoglobin-Ubx materials retain the ability of myoglobin to bind O_2_,^38^ while the immunoglobulin G (IgG) binding domains C2 and Z still bind antibodies when fused to the spider silk derivative 4RepCT.^39^ Amyloid fibrils containing chitin-binding domains bind chitin more readily than unmodified fibrils.^97^ Luciferase and xylanase both retain their enzymatic function in Luciferase-Ubx and Xylanase-4RepCT materials.^38,59^ Likewise, collagen, when fused to silk, enhances human mesenchymal stem cell (hMSC) proliferation and attachment,^98^ while addition of the VE-Cadherin extracellular domain EC1–2 to Mussel Foot Protein 5 increases adhesion of endothelial progenitor cells.^43^ Importantly, the ability to add active functions to materials is not limited to one type of ligand or material composition. Seven proteins have been successfully fused to 4RepCT and 25 proteins have been fused to Ubx (Tables 1, 2). Conversely, Enhanced Green Fluorescent Protein (EGFP) has been successfully fused to 3 different materials-forming proteins: silkworm silk light chain, Ubx, or ferritin heavy chain.^38,86,91^ Likewise, basic Fibroblast Growth Factor (bFGF) has been fused to 4RepCT, Ubx, and the silkworm silk protein light chain.^36,63,64^ This versatility in the design of fusion proteins reflects the power of this approach: in principle, any functional protein could be added to any protein-based materials, tuning the activity as well as the mechanical and physical properties of that material for a specific application.

### 3.2 Self-Assembling Proteins

The other main component in a fusion protein is the self-assembling protein. Obviously, the choice of self-assembling protein will impact the morphology, biocompatibility, degradability, and mechanical properties of the resulting materials. Self-assembling proteins can be broadly classified in three different categories: proteins that naturally self-assemble and proteins that self-assemble into materials *in vitro* but not as part of their normal function *in vivo*. Both categories have been successfully functionalized via protein fusion. In many cases, the protein sequence or conditions for assembly have been modified to optimize the production and properties of materials.^45,99−102^

#### 3.2.1 Natural Self-Assembling Proteins

Three prominent examples of proteins that naturally self-assemble to make bulk materials include silk, elastin, and resilin.^103^ Silk proteins are naturally synthesized by spiders, silkworms and bees, among others.^24,103,5^ Natural silks have a long history of use in medicine.^106,107^ These proteins are challenging to produce as recombinant monomers and to assemble *in vitro*; therefore, many laboratories have developed modified versions of silk proteins to ameliorate these problems. These modified proteins are often still referred to as the natural protein (e.g., “silk”) despite deletions and/or alterations to the protein sequence. The biocompatibility and mechanical properties of silks have made them valuable tools for many applications,^39^ including bone regeneration,^104,108^ soft tissue regeneration,^109^ cell adhesion,^40^ cardiac tissue engineering,^110^ and drug delivery.^111^

As components of the extracellular matrix, collagen and elastin have chemical and mechanical properties capable of supporting cell growth to create extensible or flexible tissues.^24,112^ Materials composed of Elastin-like Recombinamer (ELR) or Elastin-like Polypeptides (ELPs) are being developed for many applications, including skin tissue engineering and wound healing.^113^ Elastin reversibly forms hydrogels at elevated temperatures. This feature has been exploited as a facile and scalable purification technique–proteins fused to elastin or peptides derived from elastin can be separated by repeated gelation, depolymerization and washing/dilution steps.^114,115^ Consequently, a large number of proteins have been fused to elastin (e.g., Blue Fluorescence Protein,^10,50^ Cyclosporin A,^56^ and Thioredoxin^50^), although not always with the goal of functionalizing materials. Notably, proteins fused to elastin must be thermostable to withstand the temperatures used for gelation. Stabilization of the appended protein once the gel has formed may ameliorate this need.^62^

Resilin, an elastomeric protein found in insect cuticles, forms materials which are characterized by low stiffness and high energy storage capacity, making them elastic, extensible, and resilient.^19,103,116^ Resilin has important roles for flight in arthropods, leg movement in arachnids, vocalization in cicadas, and jumping in fleas.^19,117^ Resilin’s mechanical and biological properties make it a very promising scaffold for demanding but flexible tissues such as vocal cords^116,118^ and blood vessels.^119,120^ Although fewer protein fusions have been constructed using Resilin, such fusions are possible: the Kiick lab has fused several functional domains simultaneously to Resilin to create materials that bind both ligands and cells.^42^

**Figure 3:**
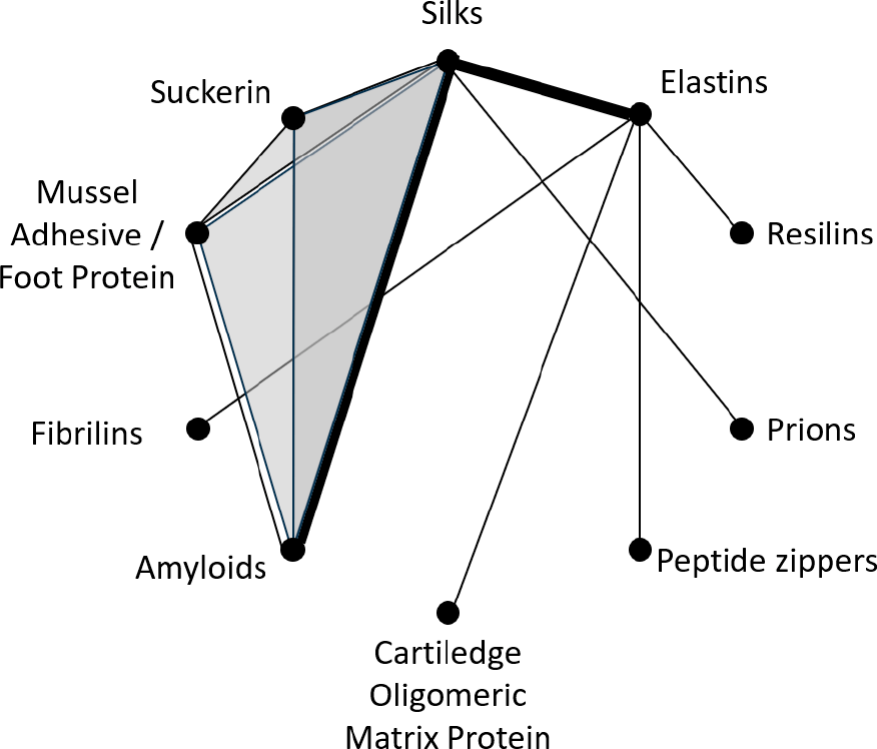
Schematic depicting the groups of self-assembling proteins that have been fused to each other to alter the structural or mechanical properties of the resulting materials.^129,130,134−150^ Each fused pair of proteins is depicted as a line, and triangles represent 3 proteins that have been combined into a single fusion protein. The weight of the lines roughly corresponds to the number of reported fusions. Suckerin is also known as squid ring tooth protein.

In addition, hybrid fusion proteins have also been created to combine the properties of silk, collagen, elastin, resilin, amyloids, and/or other proteins (Figure 3).^121,122^ Because the protein domains that determine the mechanical properties are modular, different combinations of these protein sequences can be created to optimize the mechanical properties of the hybrid material for a specific application.^95,123−127^ A diagram of self-assembling proteins that have been successfully fused to each other is depicted in Figure 3. These combination materials have recently been reviewed by Rodriguez-Cabello et al.^128^ By far the most common combination of proteins is silks and elastins, to make silk-elastin-like proteins, or SELPs. Notably, elastins typically form hydrogels, but genetically fusing the elastin sequence with a protein that forms fibers (silk protein, fibrillin, or zippers, a type of α helix) enables the formation of elastin fibrils and vesicles.^128−131^ Sometimes these fusions are created to improve the mechanical properties of the resulting materials. For instance, fibers composed of an elastin-like polypeptide and squid ring tooth protein (a.k.a. suckerin) have a breaking strength that exceeds recombinant spider silks.^132^ A similar suckerin-silk fusion has also been developed.^133^

#### 3.2.2 Proteins That Only Assemble in Vitro

Occasionally, a globular protein that does not form materials as part of its native function is able to self-assemble in vitro. Examples include the Repeats-in-Toxin (RTX) domain, a *Pseudomonas aeruginosa* alkaline protease, and Ultrabithorax (Ubx), a *Drosophila melanogaster* Hox transcription factor. The RTX domain, when fused to soluble GFP, forms green fluorescent amyloid protein fibrils and sheets, suggesting this structure may be able to host other proteins as well.^151^ A wide variety of functional proteins and peptides have been genetically fused to Ubx. Ubx self-assembles into films and fibers in vitro.^89,150^ The conditions used for Ubx assembly (aqueous buffers near neutral pH) allow proteins genetically fused to Ubx to retain their structure and activity. Full-length proteins successfully fused to Ubx include both dimers (e.g., the pro-angiogenic growth factor VEGF)^12,36^ and tetramers (e.g., fluorescent protein AmCyan).^36^ Furthermore, Ubx can form materials *in vitro* when fused to multiple proteins, such as EGFP-VEGF-Ubx or NusA-NusA-Ubx. Indeed, Ubx can accommodate fusions greater than 9 times its size (332 kDa appended protein vs 39 kDa Ubx) and still form materials.^152^

Fusion order. In theory, the functional protein or peptide can be placed at the N-terminus or C-terminus of the self-assembling protein or inserted into the middle of the self-assembling protein, most likely in a flexible region or loop.^42^ In practice, the fusion order can have a large impact on the yield of purified monomers, the ability of the monomers to self-assemble into materials, or the activity of the appended protein. For example, fusions in which the added functional protein is C-terminal to Ubx do not produce protein in *E. coli*; however, when the proteins are appended to the N-terminus of Ubx, fusions are produced that form materials and retain their activity.^36,38^ Likewise, the production levels, also termed “expression”, of four elastin-like proteins (ELPs) is higher when the functional protein is added to the N-terminus of the ELP as opposed to its C-terminus.^50^ However, the specific activity of three of the proteins, chloramphenicol acetyltransferase (CAT), thioredoxin (Trx), and interleukin-1 receptor antagonist (IL1Ra), is reduced when fused to ELP’s C-terminus, thus the function of the appended protein may also be sensitive to fusion order.^50^ Many proteins have also been fused to the N-terminus of the silk derivative 4RepCT.^39,59^ In all of the examples listed, the functional proteins retained activity in the materials. An advantage of N-terminal fusions is that stable functional proteins can help protect the nascent fusion protein from aggregation or proteolysis during translation.^153^ This benefit is especially important when the self-assembling protein contains long, intrinsically disordered (unstructured) regions, as in Ubx and some silk and elastin variants.

Other self-assembling proteins require the functional protein to be fused to the C-terminus. One example is the self-assembling protein RTX. Although GFP fused to the N-terminus of RTX domain was poorly expressed, the C-terminal fusion was easily produced and assembled into materials upon dilution or EGTA addition.^151^ Likewise, appending cold-shock protein CspB to the C-terminus of Poly(A)-binding Protein Nuclear 1 (PABPN1), an amyloidogenic nuclear protein, produced a fusion protein that was easily produced, assembled, and retained the activity of CspB in the materials.^53^ In the CBD-CsgA-Mfp3 fusion protein, the chitin binding domain (CBD) misfolds when fused to the amyloidogenic protein CsgA and a mussel foot protein (Mfp3). Altering the fusion order and the choice of mussel protein resolved this issue.^97^ While materials can be formed from a fusion of a peptide driving silica deposition (R5) to a spider silk derivative, silica deposition is more controlled when R5 is C-terminal to the silk, suggesting fusion order affects R5 folding or accessibility.^108^

A few self-assembling proteins permit fusions on either the N- or C-terminus. This ability is very desirable, since the activity of the functional protein may also be determined by fusion order. Thioredoxin can be fused to the N-terminus, C-terminus, or in the middle of elastin and still successfully form materials, although the activity of thioredoxin was never verified.^50^ No significant difference in production or assembly was observed in materials composed of either Blue Fluorescent Protein-ELP or ELP-Blue Fluorescent Protein.^50^ A hydroxyapatite binding domain, termed VTK, was fused to either the N- or C-terminus, or both, of a spider-silk inspired self-assembling protein. Although all three fusion proteins formed materials, the double fusion (VTK-silk-VTK) was better able to crystallize hydroxyapatite.^104^ All members of a family of fusion proteins containing one or more copies of Cytochrome *b*_562_ and multiple copies of an SH3 (amyloid-forming) domain were able to form amyloid fibrils. However, the morphology of the fibrils was determined by the arrangement of Cytochrome *b*_562_ sequences (N-terminal, C-terminal, or both) and the number of SH3 domains (2 or 3) in the fusion protein.^145^ Clearly, the ability of both the functional protein and the self-assembling protein to tolerate fusions to either terminus varies between proteins in a manner that is not yet predictable.

A third option is to insert the sequence of one protein into that of a host protein. This strategy requires the identification of a “cut” site on the host protein that can accept the inset without disrupting the host protein’s structure or stability. Such sites are generally surface loops or turns that make few long-range contacts within the host protein.^154^

### 3.4 Linkers

Many protein fusions require an amino acid sequence to link the self-assembling protein to the functional protein/peptide (Figure 1). The linker spatially separates the proteins, thus providing room for the two proteins to independently fold and function.^155^ Careful linker design can increase the folding, stability, expression and/or activity of fusion proteins, among other functionalities.^156,157^ The length and sequence of the linker are key factors in determining whether protein fusions are able to be produced, retain functionality, and ultimately form bioactive materials.^158^ Glycine-rich linkers provide flexibility, which may be required for each protein in the fusion to independently fold and function.^159^ Indeed, glycine is a preferred amino acid in natural linkers.^159^ Longer linkers may be required for proteins with more complicated structures, including dimers and tetramers. For example, the longer sequences were used for Ubx fusions with such proteins, GTNIDDDDKHMSGSG or GSGSH, whereas GH was deemed a sufficient linker when fusing Ubx to small soluble proteins.^36^ For natural linkers, the average linker length is roughly 10 amino acids.^159^ In these examples, glycine was used to provide flexibility, while histidine and serine increased the water solubility of the linker. Similarly, the amino acid sequence LEALFQGPNS sequence was used to link IgG and Albumin binding domains to 4RepCT.^39^ In another example, the cold-shock protein CspB, when linked to a polyalanine sequence by either GAAGG or GAA, was able to fold and form fibrils. However, the GAA sequence prevented CspB folding, and thus these materials were inactive. Therefore, the increased length and/or the enhanced flexibility of the GAAGG linker is important for creating a successful fusion of CspB to polyalanine.^53^ While most of the examples of linkers mentioned above and in the tables, are flexible, other fusion proteins may require rigid linkers for increased spatial separation of protein/peptide domains, including α-helical domains (such as (EAAAK)_n_) or proline-rich motifs.^154−156,160,161^ The amino acids Pro, Arg, Phe, Thr, Glu and Gln can act as rigid spacers. Although the fusion was not designed to form materials, the bifunctional enzyme complex Fluc-R-LRE, composed of Firefly luciferase (Fluc) and Luciferin-regenerating enzyme (LRE) fused together by a rigid linker (R, whose sequence is EAAAK), exhibits enhanced luminescence imaging, with stronger and more stable signal.^162^ Linker peptides that combined flexible, glycine-rich sequences with a rigid sequence allow the user to control linker flexibility, and thus the orientation and dynamics of the linked proteins.^116,163^

In contrast, some protein fusions have no need of a linker between different domains. This is the case for the protein fusions Ure2_1–80_-HRP, Ure2_1–93_-HRP, and Ure2_1–93_-AP composed of the HRP and AP enzymes fused to the amyloidogenic protein Ure2. These fusion proteins form amyloid nanofibrils and microgels.^143,164^ Likewise, the constructs BLP-ELP, Trx-ELP, CAT-ELP, and IL1Ra-ELP fusion proteins lack a linker peptide.^50^

### 3.5 Tags

Purification tags may also be used in some protein fusions, usually connected to the fusion protein by a flexible linker to prevent the tag from interfering with protein folding (Figure 1). A polyhistidine tag (6xHis or His_6_-tag) or FLAG-tag is often used for purification by affinity chromatography. Purification tags can be attached to either the N- or C-terminus of the functional or self-assembling protein.^104,108,165^ However, some proteins, such as Ubx, do not assemble with even short peptides or tags fused to one of their termini.^36^ Furthermore, it is important that the tag is sufficiently exposed to be able to bind the chromatography resin. Consequently, fusion order can also be important for short purification tags.^166^ The cost of subsequent purification steps used in concert with specific purification tags can vary widely.^167^ Intriguingly, due to their unique ability to withstand temperature transitions, aggregating tags such as ELPs can function both as a purification mechanism and as a self-assembling protein material.^168,169^ Depending on the net solubility of the fusion, some proteins with an aggregating tags will be expressed as inclusion bodies in bacteria, allowing for insoluble fraction collection.^170−173^ Several recent reviews on ELPs and other tag sequences are available.^167−169,174−176^

### 3.6 Typical Fusion Design Difficulties and Solutions

**Figure 4:**
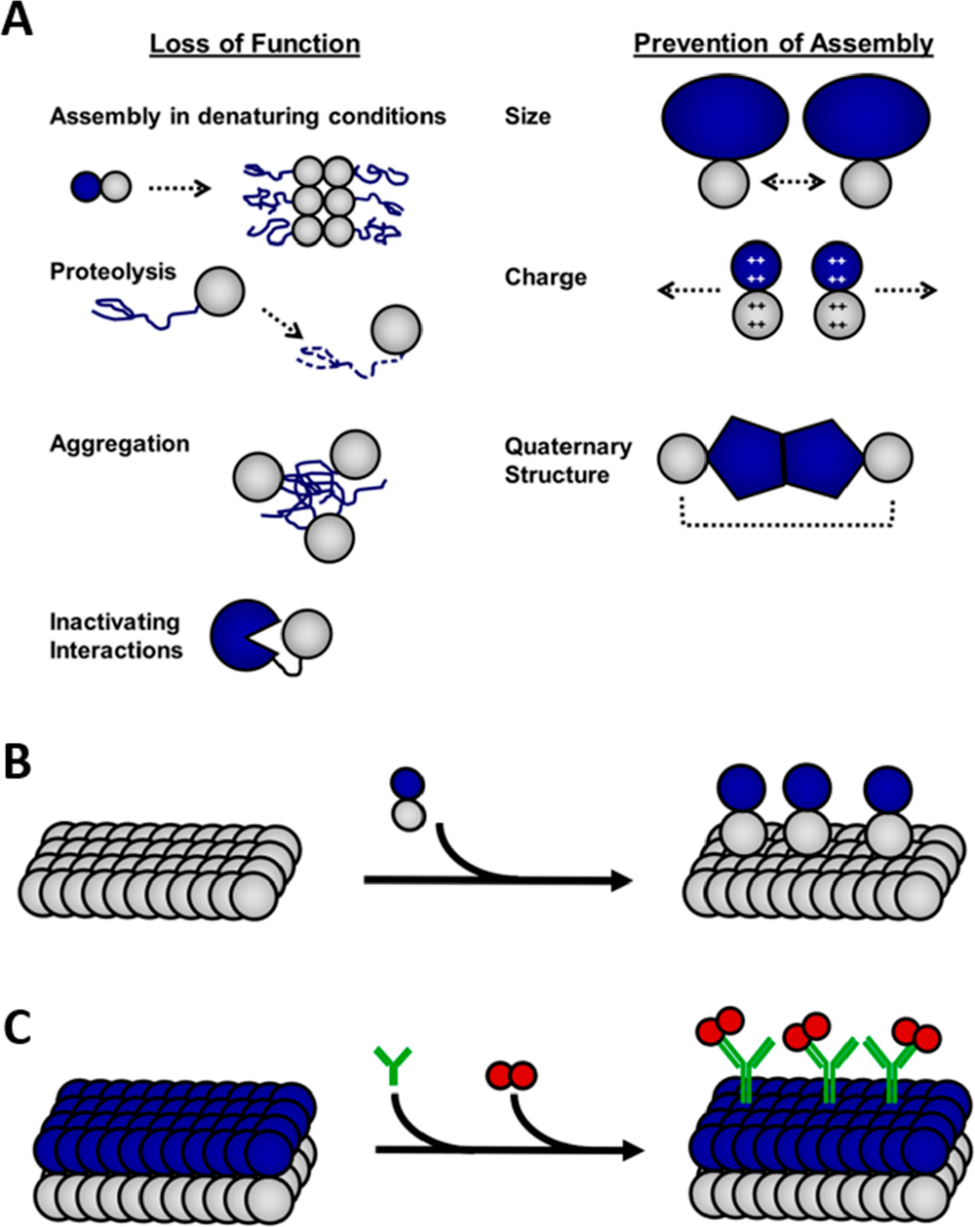
Potential problems caused by fusion proteins and noncovalent strategies for resolving fusion protein difficulties. A) Problems with fusion proteins fall into two categories, Loss of Function and Prevention of Assembly. Loss of function can be caused by the self-assembling protein (gray) inactivating the functional protein (dark blue), which in turn can lead to proteolysis or aggregation. In addition, direct binding by the functional protein to the self-assembling protein could sequester critical protein surfaces needed for either function or materials assembly. Conversely, the functional protein could prevent assembly by impairing contact between self-assembling proteins because the functional protein is too large, too charged, or because quaternary structure mispositions the self-assembling protein monomers. B) Use of unfused protein to generate noncovalent interactions between unfused protein and fusion monomers. Silk-silk interactions can bind silk chimeras bearing a functional protein (gray/blue double spheres) to a material composed only of silk (gray spheres). This approach has been used to coat the surface of silk with collagen, fibronectin, Z domain, and xylanase.^98,188^ C) Functionalization of antibodies via noncovalent interactions. Materials composed of Z-4RepCT fusions harbor a Z domain (blue component of gray/blue double spheres) which noncovalently binds the constant region of antibodies (green). The antibodies, in turn, can recognize their cognate ligand, in this case VEGF dimers (red double spheres).^39^

Despite its numerous advantages, several aspects of fusion and assembly could fail. Incorporation into materials may damage the structure of the functional protein or impede access of the molecules that bind the functional protein (Figure 3). These inhibitory packing interactions could occur within a single polypeptide chain, or between components of different polypeptide chains. Furthermore, inhibitory interactions could prevent folding or assembly of the materials-forming protein. Difficulties with generating and assembling protein fusions have prevented this technique’s widespread use (Figure 4). However, many creative solutions have been devised to overcome these issues.

#### 3.6.1 Resolubilizing Proteins from Inclusion Bodies

Methods to purify protein from inclusion bodies can perform better than standard chromatographic approaches for purification.^177^ Some self-assembling proteins (or proteins fused to aggregating tags) will express as inclusion bodies in bacteria. Most of these proteins are folded in inclusion bodies, and express in this manner due to their propensity to interact with each other. However, if a particular protein is only partially folded in inclusion bodies, it may be susceptible to degradation by proteases.^171^ This phenomenon is very protein dependent, as some proteins show increased resistance to proteases when produced in inclusion bodies (reviewed in ref (178)). A second issue is how to resolubilize inclusion bodies so that materials can be formed in a controlled manner. For proteins, like self-assembly proteins, that are folded or nearly folded, harsh denaturants may not be necessary to disrupt the inclusion bodies. Instead, optimization of the salts or cosolvents in an extraction buffer may gently resolubilize these proteins.^179−181^

#### 3.6.2 Proteolysis and Aggregation

The intrinsically disordered, or unstructured, regions of functional proteins have the potential to impact protein fusions through a variety of mechanisms. The presence of unstructured regions increases proteolysis rates,^182^ potentially inactivating the functional protein or releasing it from the self-assembling protein. In some cases, unstable or intrinsically disordered proteins are also more susceptible to amorphous aggregation, reducing the yield of soluble monomers. Finally, intrinsically disordered regions occupy more volume in solution than a structured region similar in sequence length.^182^ Therefore, disordered proteins with a shorter amino acid sequence than their structured counterparts may still exceed the size limits for fusion to a given self-assembling protein.

Finally, some proteins, especially those containing intrinsically disordered regions, may be prone to aggregation or proteolysis in their native state.^182−185^ Such aggregation could compete with the formation of orderly materials. Thus, the high protein concentrations typically required for in vitro assembly may present challenges. A list of assembly concentrations for specific self-assembling proteins is provided in Table 3.

**Table 3:** Concentrations of Self-Assembling Proteins Typically Used to Form Materials

self-assembling protein	protein concentration range for materials formation	reference
N-(+7)Ala and N-WT	300 μM	(53)
MAP	85 μM	(41)
Ure2_1–93_	50–70 μM	(164)
4RepCT	24–33 μM	(5, 39)
15mer	6–70 μM	(104)
ELP1	1–30 μM	(186)
ELP	0.72–6.2 μM	(50)
Silk light chain	0.25 μM	(63)
Mfp-5	0.1 μM	(43)
Ubx	0.05 μM	(36)

#### 3.6.3 Low Yield When Expressing the Fusion Protein

Finally, appending a functional protein has the potential to either increase or decrease the production yield of the corresponding recombinant fusion protein. Fusion to a well-folded, stable, soluble protein is a well-established strategy for improving the expression and final yield of a soluble protein of interest.^36,151^ Conversely, fusion to a desirable, yet less soluble protein will reduce protein yield. Reductions in protein solubility, and hence protein expression, caused by adding a functional protein can sometimes be corrected by adding a third, more soluble protein.^36^ As one example, adding EGFP to the VEGF-Ubx fusion protein improved protein expression without interfering with VEGF activity; both VEGF-Ubx and EGFP-VEGF-Ubx fibers were able to signal to cells.^12,36^

#### 3.6.4 Loss of Activity during Assembly

Many protein-based materials are assembled in harsh conditions (e.g., high temperature, low pH, or organic solvents), which are likely to unfold or inactivate most functional proteins.^36,38,54,186,187^ The most general solution to this problem is, when possible, to assemble materials in near physiological conditions to maintain the activity of the appended protein.^38,39^ In some cases, the sequence of the self-assembling protein can be engineered to allow assembly in conditions that preserve the activity of the fused protein.^39^

Materials assembly, even in physiological conditions, can theoretically denature or inactivate the functional protein (Figure 4A).^164^ For instance, if folding of the functional protein yields less free energy than assembly of the materials, then assembly could drive denaturation of the functional protein. If the materials-forming protein formed additional, noncovalent interactions with the functional protein that blocked its activity (e.g., physically blocked a ligand binding site), then the functional component of a fusion protein would be inactive in both the monomeric and assembled states. For example, although monomers of alkaline phosphatase (AP) fused to Ure2_1–93_ prion domain were active, reduced catalytic activity in the materials suggests the structure of AP may be damaged by assembly.^164^ In addition, *k*_cat_/K_m_ was also reduced, suggesting that the substrate access is also hindered in the materials.^164^

An alternative approach is to fold the added protein after materials assembly. Folding is generally triggered by adding an interacting ligand or cofactor. For instance, a derivative of *Nephila clavipes* spider dragline silk fused to the C-terminal domain of Dentin Matrix Protein 1 (CDMP1) was only able to self-assemble into films and form β-sheets in the presence of methanol, which would unfold many proteins.^189^ Upon incubating the assembled materials in simulated body fluid, a solution with ion concentrations similar to human blood plasma, Ca^2+^ bound to CDMP1, causing CDMP1 to fold postassembly and mineralize the films.^189^ This feature could potentially be applied in bone tissue engineering.^189^

#### 3.6.5 Fusion Impedes Assembly

The reverse problem can also occur, in which the functional protein impairs or prevents materials assembly. For instance, when the enzyme Xylanase (Xyl) was fused to a derivative of recombinant spider silk (4RepCT), the structure of the materials was unaffected; however, the assembly kinetics noticeably slowed.^59^ Moreover, the Xyl-4RepCT fusion accelerated disintegration of the material. This effect was countered by using a mixture of Xyl-4RepCT and nonfunctionalized 4RepCT and steaming the resulting foam.^59^ Highly charged functional proteins may slow or prevent materials assembly through charge–charge repulsion. Furthermore, functional proteins with quaternary structure (e.g., dimers, trimers) have the potential to mis-position the self-assembling protein and prevent materials assembly.^36^ As in the Xyl-4RepCT example, mixing unfused monomers with the functionalized protein fusion can mitigate steric clashes caused by quaternary interactions. However, multimerization of the functional protein does not always inhibit materials formation, as observed for VEGF-Ubx, SDF-1α-Ubx, bFGF-Ubx, AmCyan-Ubx, TheSSB-Ubx, TmaSSB-Ubx, and Ure2_1–93_-AP.^12,36,164^ Interestingly, Ure2_1–93_-AP proteins retain the ability to self-assemble despite the fact that alkaline phosphatase (AP) forms a dimer with mirror symmetry whereas the Ure2 dimer is axially symmetric, and no flexible linker was included to separate the two proteins.^164^ Understanding what properties of these proteins enable such versatile applications is key to improving the design of new materials.

#### 3.6.6 Steric Hindrance

A major concern in designing protein fusions is steric hindrance between the fused proteins. If the protein terminus to be tethered forms an integral part of the structure or function of that protein, then fusions to that terminus may result in misfolding, poor monomer production, reduction in ligand access to the functional protein, and/or aggregation of the fusion protein.^164,190−192^ Although the focus of this review is macroscale materials, fusions to amyloids and VLPs provide useful examples of steric hindrance. Fusion to proteins that form nanoscale materials, such as the antigen to viral coat/capsid proteins, may result in steric hindrance and protein misfolding,^193−195^ although there are many notable successes. Huang and Mason (2004) used the *Agrobacterium*-mediated transient expression system to produce and test antigen fusions formed by the Hepatitis B surface antigen (HBsAg) and GFP.^190^ When GFP is placed on the N-terminus of HBsAg, but not on the C-terminus, the protein fusion GFP-HBsAg folds correctly and assembles into VLPs. Thus, the study concludes that steric hindrance does not allow correct folding of a functional protein to the C-terminus of HBsAg, but any protein up to 239 amino acids can be added the N-terminal position.^190,196^ Likewise, fusion of GFP to Cauliflower Mosaic Virus Movement Protein changes the topology of the assembled materials due to a steric conflict between the GFP portions of the proteins.^197^ Cappelli and co-workers compared the success of fusing a β-barrel domain to 3 materials-forming proteins and 3 viral coat proteins, which form particles of varying sizes.^198^ Two of the three tested viral coat proteins formed successful fusions as well as all of the nonviral materials forming proteins.

Amyloid-forming materials may also be hindered by creating fusion proteins. This steric clash can be overcome by including extra copies of unfused protein, or altering the proteins involved. For instance, the full-length N-terminal prion domain of Ure2, which contains 93 residues, was fused to the N terminus of HRP and resulted in low enzyme activity of the amyloid fibrils. However, when the prion domain was shortened to 80 residues the catalytic activity approximately doubled. The authors concluded that the larger 93 amino acid Ure2 domain hindered substrate access to HRP.^164^ Steric hindrance can be avoided by selecting more compatible proteins for fusion, by including a long flexible linker, or by reducing the dose of the functional protein in the final material.

#### 3.6.7 An Alternative Approach to Using Fusions: Attaching Functional Proteins Postassembly

If other solutions fail, one other approach to remove problematic interactions between the functional and self-assembling proteins is to create materials using unfused protein, and then rely on noncovalent interactions between the self-assembling proteins to adhere the fusion monomers to the surface. As an example, silk–silk interactions enable recombinant silk fusion proteins to bind the surface of silk materials (Figure 4B).^98,188^ For protein fusions that are difficult to produce, this approach efficiently places the functional fusion protein only on the materials surface, where it will be most effective. A disadvantage of this approach is that surface coverage must be assessed. In a more complex design, materials were functionalized with antibodies through multiple noncovalent interactions on the material’s surface.^39^ The Z domain of Protein A, which binds the constant region of antibodies, was fused to 4RepCT silk (Figure 4C). These Z-4RepCT silk materials are capable of noncovalently binding to the constant region of a wide array of antibodies.^39^ Films and fibers of silk fusion protein Z-4RepCT, noncovalently bound to an anti-VEGF antibody, are able to specifically bind VEGF or other antibody/protein antigen pairs without the delay of additional cloning and materials production steps.^39^ Because the functionalized proteins are added after materials assembly, the danger that the materials formation process could unfold the functionalized protein is eliminated. However, adhering the functional protein to the materials via noncovalent bonds raises the possibility that the functional protein will leach from the materials at a rate determined by the dissociation kinetics of the Z domain:antibody and antibody:functional protein interactions. For applications that require the functional protein to be released, modifying these noncovalent interactions provides opportunities to tune the release rate.

**Figure 5:**
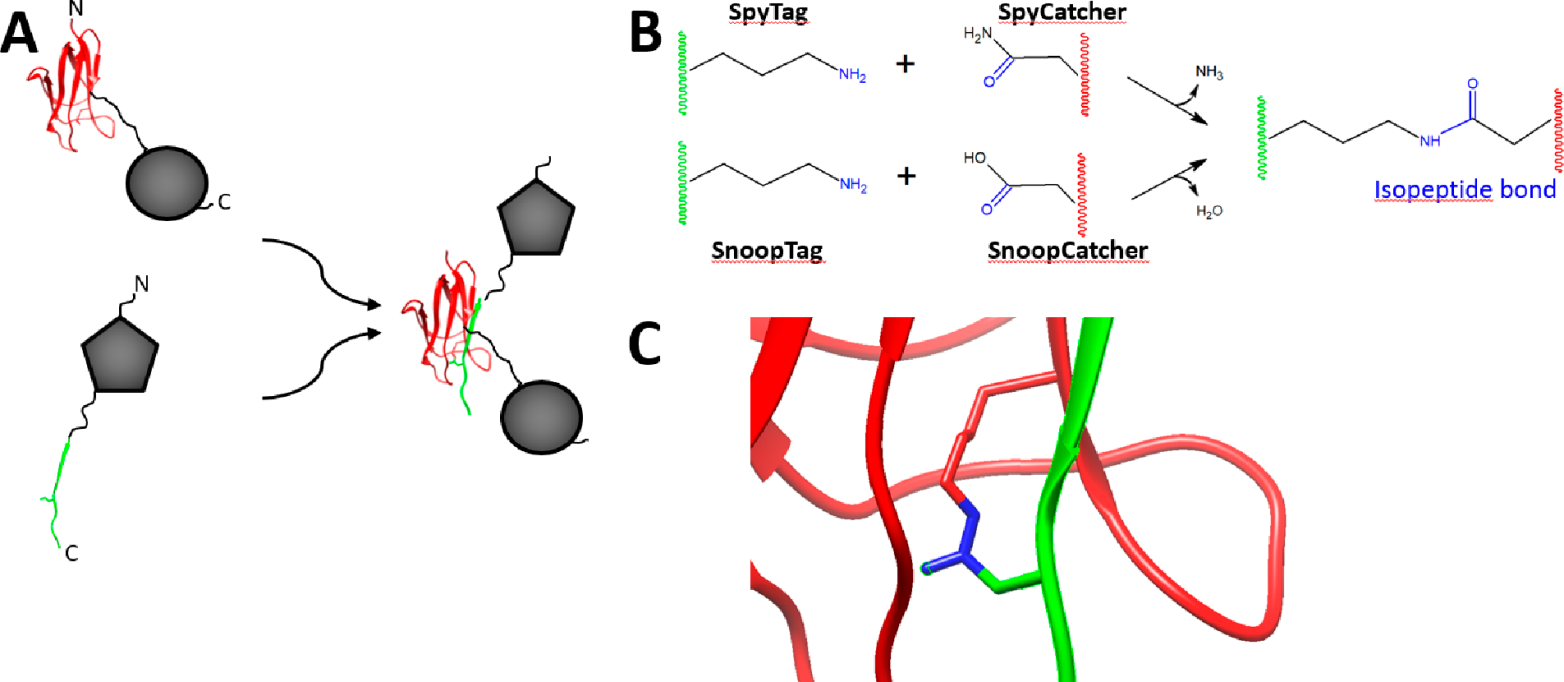
Covalent linkage of proteins via the SpyTag/SpyCatcher or SnoopTag/SnoopCatcher system. A) SpyTag (green), when genetically fused to a heterologous protein (gray pentagon) will bind SpyCatcher (red) fused to a different protein (gray sphere). After SpyTag and SpyCatcher form an isopeptide covalent bond linking the two proteins, the fused proteins (pentagon and circle) are covalently linked to each other. B) The SpyTag/SpyCatcher and SnoopTag/SnoopCatcher systems use similar amino acids to form an isopeptide bond. C) The isopeptide bond (blue) formed between SpyTag (green) and SpyCatcher (red). Figure constructed using pdb file 4MLI.^211^

An alternative to fusing the functional protein directly to the self-assembling protein is to attach the functional protein to the materials postassembly via an enzymatically catalyzed covalent bond. In the *Streptococcus pyogenes* fibronectin-binding protein (FbaB), an isopeptide bond forms spontaneously between the carboxyl terminus of one protein and the amino group of a lysine side chain. When the FbaB protein is split into two complementary pieces, the fragments, termed SpyTag and SpyCatcher, reunite and form an amide covalent bond in minutes.^199^ Each fragment can be genetically fused either to the functional protein or to the self-assembling protein. The FbaB fragments form a covalent bond that links the functional protein to the materials postassembly (Figure 5).^200,201^ Similarly, fragments of intein proteins can covalently link a functional protein to a self-assembling protein via an amide bond.^202,203^ The SpyTag/SpyCatcher system was used to attach the enzyme α-glucosidase (Ima1p) to the surface of VLPs formed by the major capsid protein VP2 from Parvovirus B19 (B19 VLPs) as a potential treatment of glycogen storage disease type II (also known as Pompe disease). The resulting materials retained enzyme activity and demonstrated enhanced stability when compared to monomeric fusion protein.^204^ Similarly, the Covid Receptor binding domain was attached to capsid-like particles using SpyTag/SpyCatcher to create an effective vaccine.^205^ The SpyTag/SpyCatcher system has also been used to enhance gelation by elastin-like proteins, including fusions in which heterologous proteins have been placed in the middle of the Elastin-Like Protein amino acid sequence.^51^ However, adding split protein fragments using SpyTag/SpyCatcher or the related systems SnoopTag/SnoopCatcher or DogTag/DogCatcher has the potential to reduce the solubility and production yield of the resulting fusion proteins, or decrease surface coverage.^201^ Thus, a new system has recently been developed using SnoopTag2, DogTag2, and Snoopligase2, in which the ∼15 kDa proteins domains are replaced with short peptides.^206^ Another alternative approach is to use self-labeling protein tags, such as HaloTag7, SNAP-tag, and CLIP-tag (reviewed in 207).^207^ These enzymes form covalent bonds with synthetic molecules, which, if attached to another protein, would create a covalent link between the two proteins. Reactive enzymes are available that create a wide variety of covalent linkages (Reviewed in 208). These enzymes have been systematically compared to facilitate selection of the optimal system for a given application.^208^ More detailed descriptions of the enzymatic ligation and affinity binding strategies for immobilizing proteins can be found elsewhere.^15,206,209,210^

## 4 Applications of Macroscale Protein-Based Materials

Protein materials can be easily optimized for many applications.^3−19,27,212^ Opportunities for specific applications arise both from the general properties of each protein material and the range of proteins that can be incorporated as protein fusions. This modular approach allows the inclusion of one or more functional peptides or proteins into a single protein sequence with tunable properties.^36,212^ Protein-based materials are being developed for a variety of biomedical and biotechnological applications such as chemical catalysis, antibody capture and immobilization, cell binding, tissue engineering/regenerative medicine, and drug delivery.^3−14^

### 4.1 Chemical Catalysis

The efficiency, stereoselectivity, and ability of enzymes to catalyze reactions in environmentally friendly conditions have increased the interest in using enzymes for industrial chemical production.^59,213^ Chemical catalysis through the immobilization and stabilization of enzymes on solid supports has many advantages, including facile separation of enzyme from the reaction mixture, which both allows enzyme recovery and aids product purification.^164^ These advantages reduce product cost, an especially important aspect in industrial processes.^59^ Although most enzyme immobilization strategies have relied on either physical entrapment, biotin/streptavidin recognition, adsorption, or nonspecific binding to a flat or microparticle surface,^213^ enzymes have also been genetically fused to proteins that form materials, including the silk derivative 4RepCT, Ubx, and the amyloidogenic protein Ure2 (Tables 2 and 3), and retained their activity once inside these materials.^36,38,59^ Another example of catalytic biomaterials includes materials based on the enzyme organophosphate hydrolase (OPH) fused to varying numbers of α-helical leucine zipper domains (H domains) and soluble linker domains (S domains), both with and without an N-terminal polyhistidine purification tag.^60^ All of the protein constructs were able to self-assemble into enzymatic hydrogels, which retained the ability to degrade organophosphate (OP) compounds, making them useful to be applied as OP biosensors or in bioremediation and decontamination systems. Furthermore, OPH-based hydrogels were capable of retaining their catalytic activity after 5 months of cold storage in buffer solution. In addition, a lyophilized protein sample was still able to form hydrogels and catalyze OP degradation upon rehydration after 6 months of storage at −20 °C. The stability of the enzyme incorporated in the materials is important for their applications to degrade organophosphate.^60^ One key challenge in multistep enzyme-mediated chemical catalysis is the need to place multiple enzymes in close proximity to limit product diffusion.^213^ Nature sometimes solves this problem by fusing together multiple enzymes that catalyze different steps in a series of chemical reactions.^214,215^ Materials composed of fusion proteins provide a unique opportunity to manipulate the geometry of enzyme packing to reduce product diffusion within the materials, and thus increase reaction yields.

The chemical environment can also be used to manipulate enzyme activity. Horseradish peroxidase, when genetically fused to an ELP, coacervates in response to osmotic stress.^216^ Interestingly, the resulting increase in substrate and enzyme concentrations accelerate the reaction rate, providing an avenue for dynamic control of the enzyme via assembly and disassembly.^216,217^

### 4.2 Antibody Capture and Immobilization

Many applications are based on functionalization of materials with affinity domains that recognize antibodies. Once antibodies noncovalently bind these materials, the materials can be potentially used to bind specific ligands (as in sensors),^41^ to bind and display proteins (for tissue engineering applications),^39^ or to deliver proteins (for therapeutics).^39,44^ In this versatile approach, the same base material can be used with any antibody/antigen combination. In particular, effective immobilization of antibodies on a solid support is of extreme importance for the specificity and sensitivity of immunoassay procedures.^41,218^ For instance, recombinant mussel adhesive protein (MAP), when genetically fused with the B and C domains of the immunoglobulin G (IgG)-binding Protein A, enabled efficient and oriented antibody immobilization onto diverse surfaces such as glass, polystyrene, and aluminum.^41^ This functionalized material has potential applications in immunoassays and immunosensors.^41^ As discussed above, the Z domain of protein A, which binds IgG, has been genetically fused to the silk derivative protein 4RepCT,^188^ The resulting materials were used to immobilize the growth factor VEGF, which is largely used in cell culture and tissue engineering applications. These versatile materials could potentially be applied to immobilize other specific proteins depending on the type of antibody captured.^39^

### 4.3 Cell Binding

Many materials have been modified via peptide fusion to improve binding to specific types of cells (Table 1).^219−221^ Integrin- and fibronectin-binding sequences were both inserted into the collagen-like protein Scl2 from *Streptococcus pyogenes* fused to recombinant silk, thus creating chimeric collagen-silk fibers, allowing these materials to attach and grow human mesenchymal stem cells (hMSCs).^98^ Fibronectin is a component of the extracellular matrix (ECM) that binds to many molecules, including other ECM proteins and cell adhesion molecules, and is composed of subunits termed Type I, Type II, and Type III.^221^ Recombinant silk-elastin-like proteins (SELPs) blended with 6mer+FNII, which is a sequence repeat from spider silk genetically fused to fibronectin type II, formed films with increased ability to promote cell adhesion of human skin fibroblasts when compared to 6mer+FNII alone.^40^ Likewise, coating a plastic surface with a fibronectin-silk variant (FN-4RepCT) improves the seeding efficiency of dermal microvascular endothelial cells.^222^ The great variety of peptides that recognize specific types of normal and diseased cells^223^ have the potential to increase cell attachment and migration to materials for applications involving diverse tissues.^224^ These tools make peptide fusion materials a particularly powerful tool for cell analysis (cell isolation, detection) and tissue engineering (cell attachment and patterning) applications.

### 4.4 Tissue Engineering

Protein-based materials are widely applied in tissue engineering and regenerative medicine (reviewed in refs (223)–^225^).^40,64,104,106^ Because cells respond to both mechanical and chemical cues, matching the materials’ properties to the target tissue is very important for tissue regeneration or engineering. The sequence of recombinant self-assembling proteins can be easily modified to alter the structural or mechanical properties of the resulting materials using standard molecular biology techniques, as in hybrid materials composed of silk elastin and collagen sequences.^40,121,123,124−127,226−231^ This plasticity allows the properties of the materials to be tailored to suit a variety of applications.^30^ A large number of proteins and peptides function as chemical cues that control cell behavior and support cell growth, attachment, and proliferation within the scaffold. In addition to peptides, growth factors (generally dimeric proteins) have also been fused to materials to direct cell behavior. For instance, VEGF-Ubx fibers can induce migration, attachment, and survival of human umbilical vein endothelial cells (HUVECs).^12^ A double-layered adhesive microneedle bandage was able to preserve cardiac muscle and enhance regeneration during rat heart remodeling. These bandages were composed, in part, of Mussel Adhesive Protein fused to peptides derived from VEGF and fibronectin.^232^ Recently, the Xia lab fused PDGF-BB to silk sericin to create hydrogels that both augmented osteogenesis by mesenchymal stem cells and inhibited cell differentiation into adipocytes.^227^ Despite lacking a folded protein structure, peptides can be surprisingly potent. An ELP, modified with peptides to promote laminin adhesion and hydroxyapatite binding, forms osteoid tissue and enhances bone healing in vivo.^233^ Such materials have the potential to be used not only as scaffolds for tissue engineering, but also as printable bioinks, allowing spatial control of cell function during 3D tissue reconstruction.^234^

Many protein biomaterials have been engineered to induce biomineralization and aid in bone regeneration.^66,104,235,236^ A recombinant silk derivative fused to bone sialoprotein (BSP), termed 6mer + BSP, formed films that retained the capacity of BSP to induce deposition of calcium phosphate, which is important for bone mineralization.^66^ Films formed by 6mer alone did not induce mineral deposition. Furthermore, 15mer recombinant silk separately fused to the N-terminus, C-terminus, or both termini of the hydroxyapatite binding domain VTK increased the formation of crystalline hydroxyapatite and allowed growth and differentiation of hMSCs.^104^ In a final example, a transgenic silk fibroin modified with a D-rich peptide has a higher Ca^2+^-binding activity *in vitro* and presented higher activity and mineralization capacity than natural silk fibroins.^37^

### 4.5 Drug Delivery

**Figure 6:**
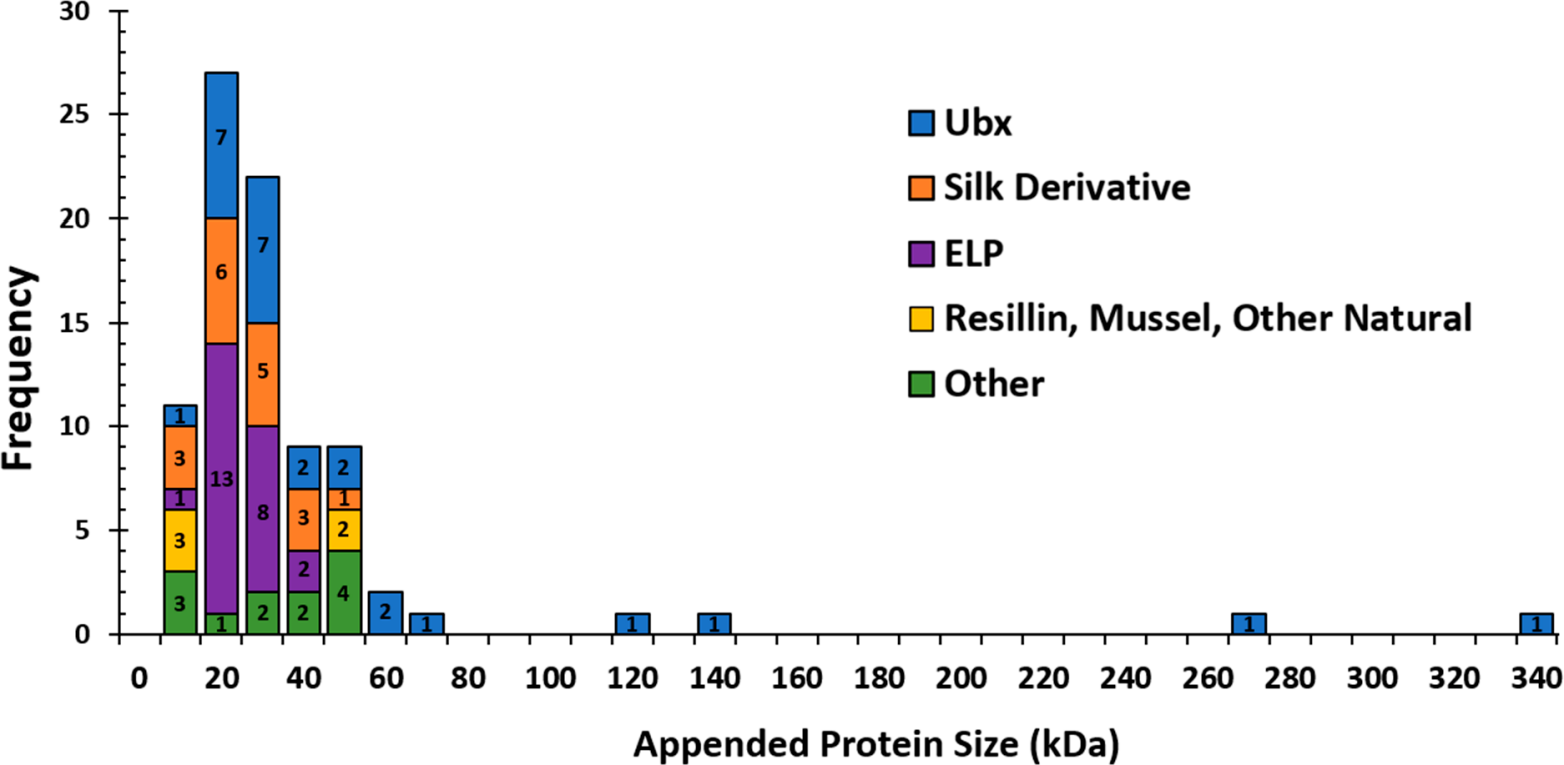
Analysis of the size fusion proteins reported in Tables 1 and 2. The identity of the self-assembling protein in each fusion is indicated by color.

Protein-based materials also have the potential to be used as drug delivery systems.^24,26^ Genetically engineered biomaterials that are biocompatible, biodegradable, and bind ligands with high specificity have the potential to be used for the controlled release of drugs and other molecules.^5,124,237^ Recombinant lipid-ELP biomaterials have been developed to self-assemble into drug delivery micelles of different sizes and shapes, such as spheres and rods (Figure 6). The yeast N-myristoyltransferase enzyme was coexpressed with ELP in *E. coli* followed by a post-translational modification of ELP with myristic acid. Myristoylated ELPs can thus form micelles with lipid cores that can encapsulate hydrophobic anticancer drugs, such as doxorubicin and paclitaxel.^238^ NGR-ELP_BC_ micelles were constructed to target tumors by fusion of ELP block copolymers, consisting of both hydrophilic and hydrophobic ELP blocks, and NGR, which targets the CD13 membrane protein in angiogenic blood vessels in tumors.^239^ Additionally, the fusion of the p53-interacting anticancer protein, azurin, to ELP resulted in the formation of micelles at temperatures higher than the lower critical solubility temperature.^80,170^ The resulting micelles were found to have six times higher apoptotic induction in LNCaP prostate cancer than the azurin-ELP monomers, indicating an effective delivery system for targeted cancer treatment.^80^

Variation in the number and ratio of silk and elastin motifs in SELPs tunes the aggregate size, stability, and thermoresponsive behavior in nanogels composed of these proteins.^121^ Importantly, the ability to tune the physicochemical properties of silk nanogels alters the pore size, which in turn dictates the diffusion of molecules through hydrogels.^240^ Additional structures and physical properties have been achieved by combining ELPS with synthetic polymers.^231^

### 4.6 Enhancing Vaccine Efficacy

Although the focus of this review is macroscale materials, important lessons can be learned from protein fusions used to create nanoscale particles for vaccines. Fougeroux et al. demonstrated that displaying the Receptor Binding Domain of SARS-CoV-2 Spike protein when generically fused to capsid-like particles, elicited a much more robust immune response than free Receptor Binding Domain protein, highlighting the benefits of creating particles with a high density of the antigen.^205^ Indeed, increasing antigen density by fusing to capsid-like particles or VLPs have increased the immunogenicity of other antigens as well.^241,242^ This display of multiple antigens both elicits a broader response, and improves cell uptake.^194,195^ Therefore, viruses may be much more amenable to this potent approach to materials functionalization than generally assumed. Other proteins that form nanoscale materials also see improved antigenicity, including hemagglutinin-ferritin and flagellin fused to several epitopes from SARS-CoV-2.^243,244^ These increases in cell attachment and cell response appear to occur in macroscale materials,^12^ although far fewer quantitative studies have been reported.

## 5 Conclusions and Future Perspectives

### 5.1 Summary

Each protein-based material has a unique set of characteristics that define its optimal applications, such as biocompatibility, surface chemistry, and mechanical properties. As the number and diversity of protein-based materials grows, the opportunity to match or engineer the properties of a material for a specific application improves dramatically. Applying the protein fusion technique to these proteins allows creation of covalently functionalized materials in a single pot, single component, single step reaction. For each self-assembling protein, the design of protein fusions will be limited by factors, such as protein size, extensibility, stability, and fusion order, that are unique for that material. Nevertheless, the number of materials that have been functionalized by protein fusion has expanded in the past decade, creating new opportunities for materials engineering and functionalization. Figure 6 depicts all of the fusion protein materials discovered through an extensive literature search, as a function of the appended proteins’ size. Clearly, while incorporated proteins vary in size and complexity, most successful fusion proteins are monomers that are <50 kDa in size.^206^ Mixing fusion proteins with unfused proteins in materials may allow larger proteins to be incorporated using existing materials.^245^ These successes have also revealed gaps in knowledge and in the currently available techniques that limit this field.

### 5.2 Materials Structure and Assembly Mechanisms

**Figure 7:**
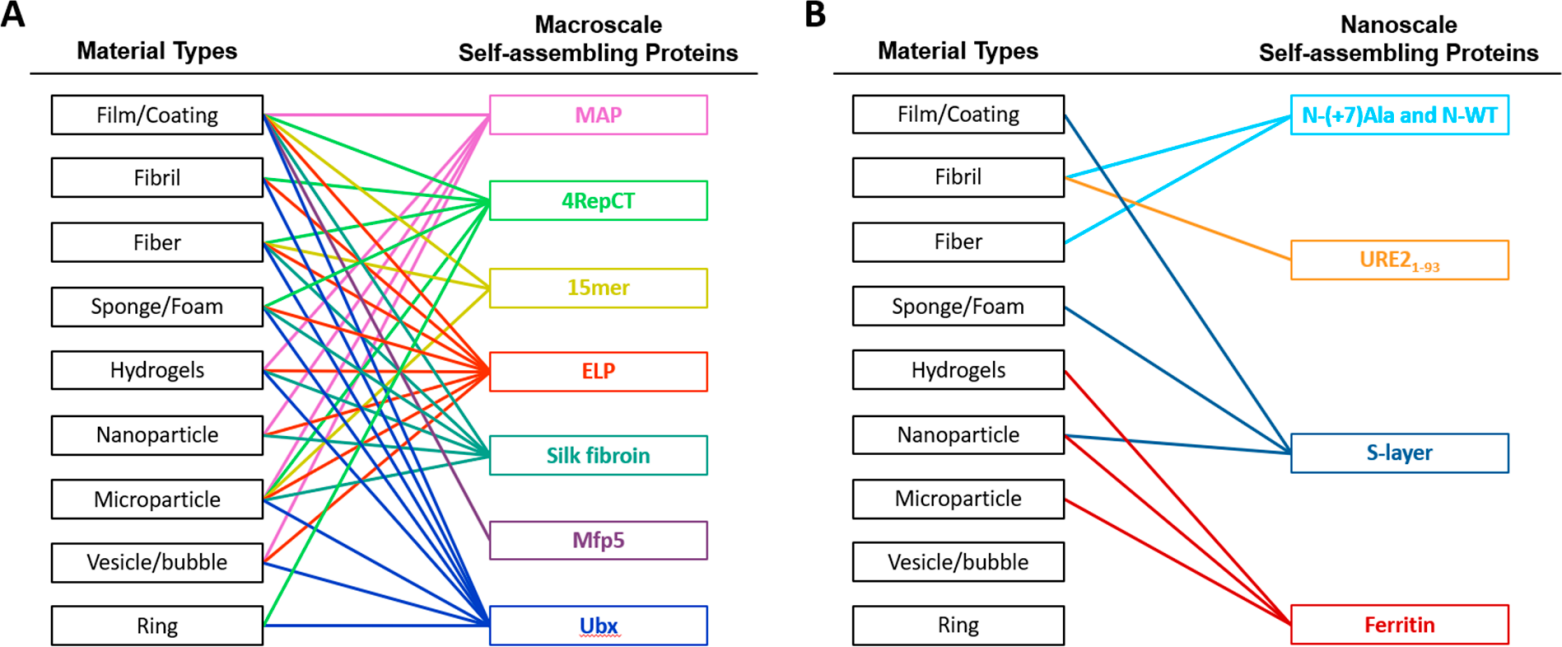
Materials formed using self-assembling macroscale and nanoscale proteins. A) Macroscale materials are linked to which types of materials have been formed. Generally, most macroscale materials can form more different morphologies of materials.^36,39,48,123,150,152,219,246−266^ B) Nanoscale materials have been linked to material types which have been formed.^58,68,267−272^

Compared with proteins that form nanoscale materials, self-assembling proteins that are composed of many interaction motifs interspersed with flexible linking regions–such as silk, elastin, and Ubx - are capable of forming the most different types of macroscale materials (Figure 7). For many macroscale materials, little is known about the mechanisms of monomer assembly, or the structure and protein interfaces that form the final product. Understanding these mechanisms for a wide variety of protein systems should improve both the design of new self-assembling proteins and facilitate optimization of existing materials.^246^ These traits have been explored for many proteins that produce nanoscale materials, such as virus-like particles or capsid proteins, bacterial S-layer proteins, and amyloids. Principles learned from these well-characterized nanomaterials may guide understanding of their macroscale counterparts.

### 5.3 Understanding Phase Behavior/Multiphase Behavior

Because protein assembly is driven, at least initially, by noncovalent interactions, the physical and chemical environment can have a substantial impact on materials formation. Thus, phase behavior is an inherent property of self-assembling proteins, and a deeper understanding of how to add or eliminate the dependence of assembly on a particular parameter is needed to design new, sensitive protein systems and to enable their reliable use in many applications.^273^ In particular, a prominent characteristic of many ELPs is multiphase behavior, in which monomers respond to many stimuli and/or have multiple temperature transitions.^273−275^ Because each phase transition is sequence-dependent, fusing two ELPs together, each with a different sequence, will create a protein with multiple transitions, and thus multiple phases.^275^ This idea is extended in artificial IDPs designed by the Chilkoti lab, which designed two proteins, one an artificial PGXG-repeat ELP, and the other a partially ordered polymers (ELP with polyalanine helices inserted within the sequence). Mixtures of these proteins create tunable, complex unique microarchitectures using only droplet microfluidics and heating/cooling, both scalable approaches.^274^ The ability of multiphasic sequences to respond to the environment allow the creation of new 3D architectures without having to change the sequence or the assembly method. The function of these materials, and thus their potential applications, is determined by the size, shape, and internal architecture of the materials.^274^ These very attributes create unique challenges for these materials. First, once the desired material has been created, many applications would require the material to maintain its shape through transfer to other solvents or temperatures required for storage, shipping, or in its final application. Such changes elicit structural transitions in first-generation materials. Second, many applications require predictable behavior, so that the materials only respond to a specific desired event (e.g., ligand binding), and not to other factors such as changes in pH, ion concentration, or electric potential that can occur *in vivo*.^273^ One answer to this design issue is to use unnatural amino acid incorporation to photo-cross-link the materials, thus trapping them in the preferred form.^274^

### 5.4 Predicting Fusion Success

Not all protein fusions are successful. As discussed above, the production and assembly of materials-generating proteins can only accommodate a fused functional protein on the N- or C- terminus. In addition, not all functional proteins are equally good candidates for fusion to a self-assembling protein. An interesting example is fusing the LOO-GFP family of proteins to Ubx. LOO-GFPs are biosensors in which OPT GFP has been circularly permuted and then terminally truncated to remove single β-strand from the GFP barrel. Different LOOs, with either strand 7, 8, or 9 missing, were fused to Ubx and expressed together with their missing peptide in *E. coli*. Large differences in protein accumulation for the different LOO-Ubx variants were observed, even though the appended proteins were nearly identical and linkers were used to reduce steric hindrances during folding.^245^

There is no obvious way to predict whether an N-terminal or C-terminal fusion is likely to be more successful. Based on the data collected in Tables 1 and 2, roughly twice as many N-terminal fusions have been constructed (50) relative to C-terminal fusions (27), both of which far outstrip fusions in the middle of the sequence (4) or to both termini (5). However, these differences likely reflect both protein capability and bias in fusion design. A meta-analysis of the existing information for macroscale proteins might reveal unexpected correlations between local sequence properties and success rate. Although many successful fusions with nanoparticle-forming proteins have been reported, a large number of scientists in this field report unsuccessful fusions. One possibility is that the fusion success rate is higher for proteins that form macroscale materials because these proteins often have intrinsically disordered regions, which both provide the flexibility required to avoid steric clashes and the extensibility required to accommodate the appended protein within the 3D structure. In support of this idea, the Ubx protein cannot tolerate fusions to its structured C-terminus, but can support many diverse fusions to its intrinsically disordered N-terminus (Table 2).

### 5.5 Patterning

Many applications may require the functional proteins to be patterned within the materials. For instance, tissues are composed of many cell types, and thus the proteins that instruct each cell type, as well as the target cells, will ultimately have to be patterned within the material. Similarly, the specificity and reliability of biosensors can be increased by multiplexing many sensing proteins that each bind a different single ligand. Techniques frequently utilized to produce silk-based scaffolds for tissue engineering can potentially be applied to create new micro- and nanoscale patterned silk materials, including nanoimprinting and 3D printing.^276^ Ubx-based materials can also be patterned to produce microscale spots, stripes, and gradients without the need of additional equipment or solution conditions that could harm the functional protein.^38^ Developing more general and versatile techniques for patterning proteins would significantly expand the number of protein materials that could be used in applications requiring patterning.

### 5.6 Ligand Diffusion

For materials designed to bind and/or deliver soluble ligands, the rate of ligand diffusion through the materials will impact binding and release kinetics. The structure, porosity, pore connectivity, surface chemistry and degradation rate of each material is expected to have a substantial impact on ligand diffusion;^277−280^ however, little is currently known about the extent to which different materials permit or exclude diffusion of different types of ligands in protein-based materials. In one of the few studies in this area, Baxa et al. demonstrated that substrate diffusion through filaments only slightly reduces enzyme activity.^281^ The diffusion of molecules through nonprotein hydrogels has been more thoroughly studied.,^240,245,276−280,282,283^ The main factors affecting diffusion rate include hydrogel pore size and electrostatic interactions between the hydrogel and the diffusing molecules.^240^ These hydrogel studies provide a useful guide for investigation of diffusion through protein matrices. The more chemically and topologically complex nature of a protein is expected to make similar studies in proteins complex, yet very interesting.

### 5.7 Guest Protein Dynamics

Dynamics are a key aspect of the behavior of many proteins. For instance, enzyme motions often accompany chemical catalysis.^284^ Other proteins also require large-scale motions as part of their function.^285^ How does incorporation into materials impact the ability of these proteins to move and thus function? To what extent, if at all, is protein motion restricted in different materials? Can the sequence of a self-assembling protein be altered to enhance or suppress motions in a fused functional protein?

### 5.8 Fusion Effects on Materials Properties

Little is known about the impact of fusions on the properties of protein-based materials. In most cases, materials assembly and structure is generally only verified by microscopy. Thus, the impact of fusions on assembly kinetics, protein structure, and mechanical properties of the host material are often ignored. However, fusion to EGFP decreases the extensibility and increases tensile strength of Ubx materials,^36^ and the structural features of proteins fused to amyloids impact both the assembly and Young’s modulus of the resulting materials.^97^ Forman et al. demonstrated that the conformation and position of proteins displayed on amyloid fibrils impact fibril morphology.^145^ Conversely, the materials could alter the function of the appended protein. For instance, interactions between the LOO8 biosensor protein and Ubx materials hampered analyte binding, a problem that was largely mitigated by altering the buffer.^245^ These results highlight the need to better investigate the ability of fused proteins to impact the properties of materials and vice versa.

### 5.9 Interfaces with Nonprotein Materials

Finally, several applications may require functionalized protein-based materials to interface with other types of materials. In tissue engineering, interfaces between tissue types (e.g., bone/tendon) may be required to rebuild damaged bodies. Likewise, sensors must not only bind ligand, but also detect when ligand is present. For many approaches, this will require sensors to stably bind detection devices such as a quartz-crystal nanobalance.^41^ New proteins may need to be designed or evolved to meet these needs.

Addressing these issues in any material will substantially increase the utility of all protein-based materials. In addition, each protein-based material exhibits a unique set of beneficial attributes and engineering challenges. Identifying new self-assembling proteins amenable to protein fusion and defining the range of proteins that can be successfully fused will expand the toolbox, allowing materials to be selected that better match the needs of each application. Finally, developing creative assembly techniques has the potential to incorporate difficult proteins that cannot be immobilized by standard single-component approaches.^39,286^

## Abbreviations

ABD: Albumin-binding domain
AP: Alkaline Phosphatase
BFP: Blue Fluorescent Protein
bFGF: basic Fibroblast Growth Factor
BSP: Bone Sialoprotein
CA: carbonic hydrase
CABP: Calcium-binding protein
CAT: Chloramphenicol Acetyl Transferase
CDMP1: C-terminal domain of Dentin Matrix Protein 1
CspB: Cold Shock Protein from *Bacillus subtilis*
CXCR4: C-X-C Chemokine Receptor Type 4
EBFP: Enhanced Blue Fluorescent Protein
EGFP: Enhanced Green Fluorescent Protein
ELPs: elastin-like proteins
ELR: Elastin-like Recombinamer
ECM: extracellular matrix
FbaB: fibronectin-binding protein
FL: silk fibroin light chain
FN: Type III 8–10 domains of fibronectin
FNII: sequence from Fibronectin Type II
GFP: Green Fluorescent Protein
GST: Glutathione S-transferase
hMSCs: human mesenchymal stem cells
HRP: Horseradish
HUVECs: human umbilical vein endothelial cells
IgG: Immunoglobulin G
IL1Ra: Interleukin-1 Receptor Antagonist
LIF: Leukemia Inhibitory Factor
MAP: mussel adhesive protein
MBP: Maltose-binding Protein
MFP: Mussel foot protein
MPH: methyl parathion hydrolase
NusA: N utilization substance protein A
OP: Organophosphate
OPH: Organophosphate Hydrolase
PABPN1: Poly(A)-binding Protein Nuclear 1
PFK: Phosphofructokinase
RTX: Repeats-in-Toxin
SDF-1α: Stromal cell-derived Factor 1α
SELPs: Silk-elastin-like Proteins
SUMO: Small Ubiquitin-like Modifier protein
TneSSB: *Thermotoga neapolitana* single-stranded DNA binding protein
TmaSSB: *Thermatoga maritime* single-stranded DNA binding protein
Trx: Thioredoxin
Ubx: Ultrabithorax
VEGF: Vascular Endothelial Growth Factor
VLP: Virus-like particle
Xyl: Xylanase
